# Supplementary figures and images for: SABMIS: sparse approximation based blind multi-image steganography scheme
Source: PeerJ Comput Sci. 2022 Nov 28;8:e1080. doi: 10.7717/peerj-cs.1080 (PMC9748825; doi:10.7717/peerj-cs.1080)

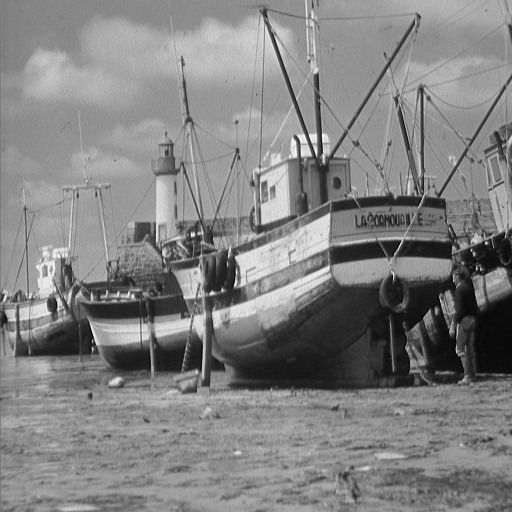

Supplement: Supplemental Information 1 — All images used for experiments and respective licenses [file peerj-cs-08-1080-s001.zip › ImagesFiles/boat.png]

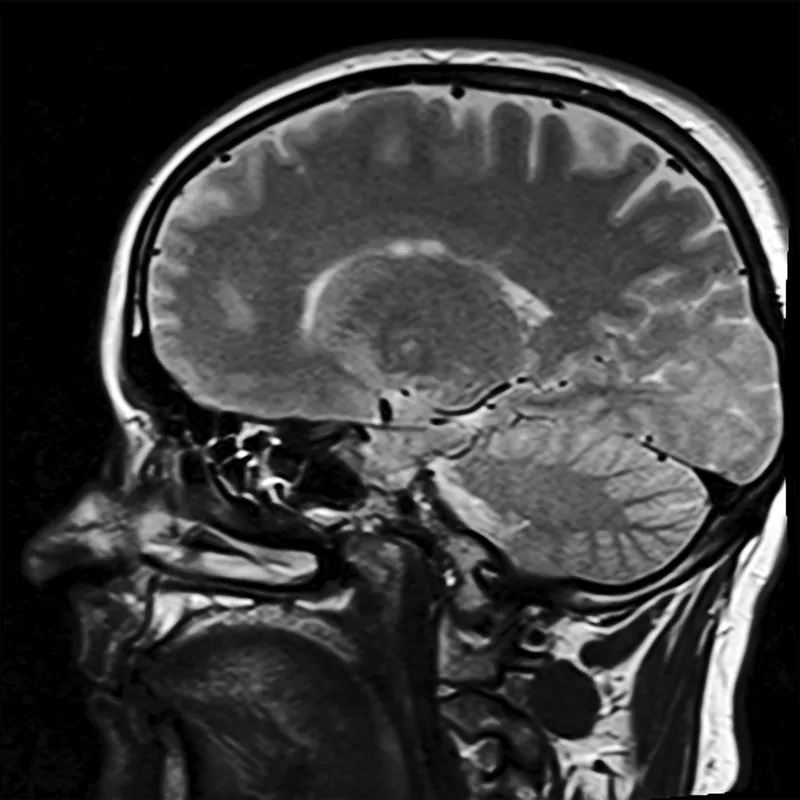

Supplement: Supplemental Information 1 — All images used for experiments and respective licenses [file peerj-cs-08-1080-s001.zip › ImagesFiles/BrainImage.bmp]

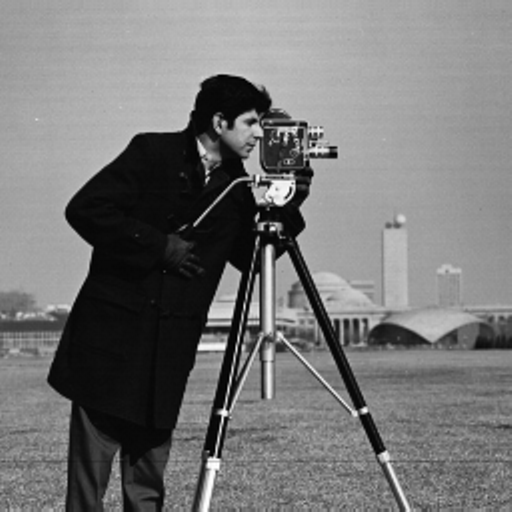

Supplement: Supplemental Information 1 — All images used for experiments and respective licenses [file peerj-cs-08-1080-s001.zip › ImagesFiles/cameraman.tif]

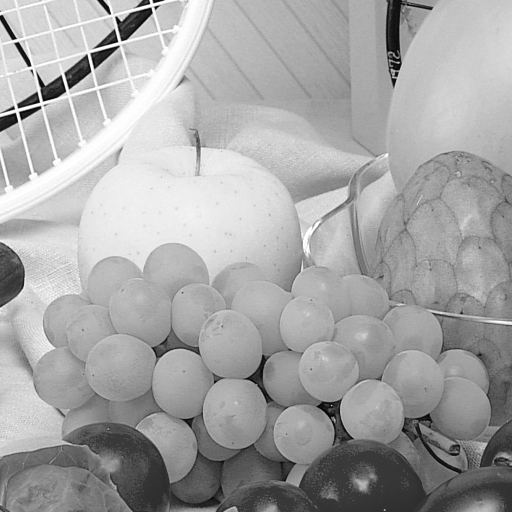

Supplement: Supplemental Information 1 — All images used for experiments and respective licenses [file peerj-cs-08-1080-s001.zip › ImagesFiles/fruits.png]

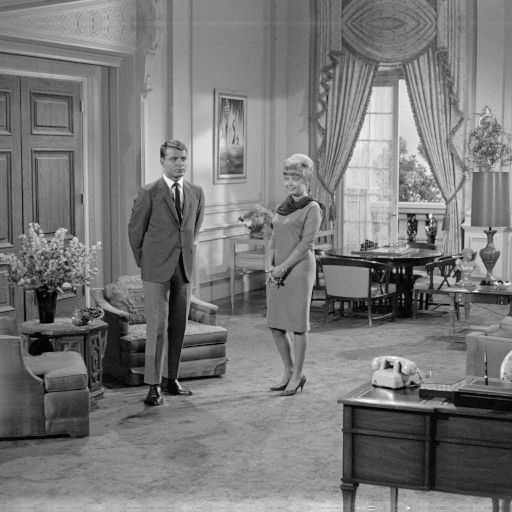

Supplement: Supplemental Information 1 — All images used for experiments and respective licenses [file peerj-cs-08-1080-s001.zip › ImagesFiles/livingroom.tif]

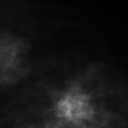

Supplement: Supplemental Information 1 — All images used for experiments and respective licenses [file peerj-cs-08-1080-s001.zip › ImagesFiles/Mammogram1.png]

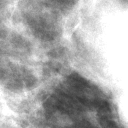

Supplement: Supplemental Information 1 — All images used for experiments and respective licenses [file peerj-cs-08-1080-s001.zip › ImagesFiles/Mammogram2.png]

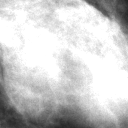

Supplement: Supplemental Information 1 — All images used for experiments and respective licenses [file peerj-cs-08-1080-s001.zip › ImagesFiles/Mammogram3.png]

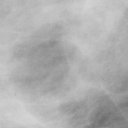

Supplement: Supplemental Information 1 — All images used for experiments and respective licenses [file peerj-cs-08-1080-s001.zip › ImagesFiles/Mammogram4.png]

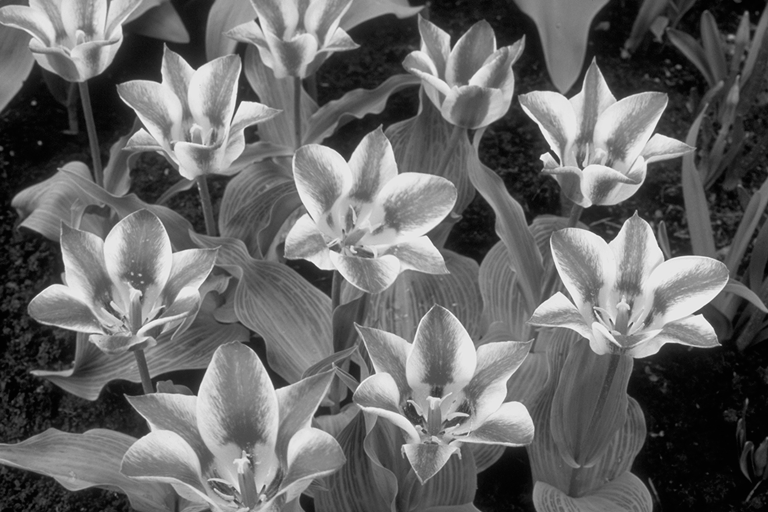

Supplement: Supplemental Information 1 — All images used for experiments and respective licenses [file peerj-cs-08-1080-s001.zip › ImagesFiles/tulips.png]
